# Positive Psychology Interventions in Patients With Prostate Cancer: A Systematic Review

**DOI:** 10.1002/cnr2.70494

**Published:** 2026-02-12

**Authors:** Eleanor Xu, Quan H. Phung, Karie Runcie, Michael A. Liu

**Affiliations:** ^1^ Department of Clinical Psychology University of Houston Houston Texas USA; ^2^ Department of Hematology and Oncology Mary Washington Healthcare Fredericksburg Virginia USA; ^3^ Division of Hematology and Oncology Columbia University Irving Medical Center New York New York USA; ^4^ Robert H. Lurie Comprehensive Cancer Center, Northwestern University Feinberg School of Medicine Chicago Illinois USA

**Keywords:** mindfulness, positive psychology, prostate cancer, quality of life, resilience

## Abstract

**Background:**

Prostate cancer is the most common malignancy in men and may often result in psychiatric symptoms due to the direct disease effects, hormonal treatments, functional losses, and psychological responses of patients to the cancer. Positive psychology interventions have shown promise in alleviating psychological symptoms in patients with chronic diseases but are infrequently studied in patients with prostate cancer.

**Aims:**

Our systematic review aimed to examine the benefits of positive psychology interventions for patients with prostate cancer.

**Methods and Results:**

We searched the PubMed/Medline, Embase, and PsycInfo databases yielding 1078 initial studies, 10 of which met inclusion criteria. The methodological quality of included studies was formally assessed. Positive psychology interventions consisted of mindfulness‐based therapies, meditation, hope and resilience therapies, and well‐being therapies. Most studies showed a positive effect on outcomes for patients with early to advanced stage prostate cancers, including psychological distress, mood disorders, anxiety, quality of life, happiness, and life satisfaction. However, most studies were limited by small sample size, qualitative findings, and loss to follow‐up.

**Conclusion:**

This systematic review demonstrates that positive psychology approaches may have benefit in patients with prostate cancer. Hormonal changes and inflammatory biomarkers are potential pathways through which positive psychology interventions influence this population.

## 1 Introduction

Prostate cancer is the most common malignancy in men and the second leading cause of male cancer‐related mortality, encompassing around 11% of cancer‐related deaths [1]. Recent advances in prostate cancer treatment as well as improved access to treatments have been correlated with an overall global reduction in mortality [2]. However, prostate cancer and its treatments may contribute to psychological consequences; for example, mood disorders can be common yet underrecognized and are associated with increased costs and healthcare utilization [3]. Multiple factors contribute to psychiatric symptoms in patients with prostate cancer including: lower testosterone from hormonal treatment, direct disease effect, functional losses such as erectile dysfunction, and the psychological response to having a cancer diagnosis. Subsequently, there has been promising evidence that psychotherapeutic support and interventions can significantly enhance the well‐being of patients with prostate cancer [4].

Positive psychology is a relatively new domain of psychology that has not been well studied among patients with cancer [5, 6]. At its core, positive psychology focuses on the pursuit of happiness and positive attitudes towards individual experiences and traits as the goal of psychotherapy. Positive psychology interventions aim to empower individuals by enhancing their ability to cope with and reinterpret negative emotions or challenges rather than eliminating suffering. One of the major goals is to teach people to substitute negative emotions with positive ones, which can have preventive, therapeutic, and physiological effects and build optimistic beliefs that can serve as a protective factor against illness [7]. There is evidence that positive emotions may improve productivity, relationships, and physical health, in addition to relieving depression [8, 9]. Proposed interventions include well‐being therapy, quality of life therapy, positive psychotherapy, mindfulness, and strength‐centered therapy [10, 11, 12, 13]. It has been demonstrated that a state of positive health may affect longevity and prognosis in a spectrum of chronic disorders such as cardiovascular disease, stroke, and HIV [7]. Seligman describes the optimal diseases to study positive psychology should include characteristics such as: variable prognosis, serious enough to affect longevity, costly in medical care, disabling, and a prevalent public health problem [7]. Prostate cancer, therefore, is a prime target of study; however, the potential impact of positive psychology in this disease remains unclear. Our study aim is to conduct a systematic review on the benefits and therapeutic effects of positive psychology interventions in patients with prostate cancer.

## 2 Methods

### 2.1 Search Strategy and Study Selection

The systematic review was conducted according to the PRISMA guidelines (Preferred Reporting Items for Systematic Reviews and Meta‐Analyses). A systematic literature search was conducted using the PubMed/Medline, Embase, and PsycInfo databases using a combination of MeSH terms and free text words, including publications up to April 2025. Inclusion criteria consisted of original research studies and studies in the English language; reviews, letters, editorials, and case reports were excluded. Studies were eligible if they included at least one positive psychology therapy intervention and the title or abstract mentioned the inclusion of patients with prostate cancer (no restrictions based on age, number of subjects, or stage of disease). When available, subject headings with MeSH terms were used in the search strategy, combined with free text words. Descriptors are listed in the search phrases (see Supplement) based on a list of positive psychology relevant keywords and interventions. Abstracts of the publications were screened for by two individuals, who independently reviewed reports identified in the search to meet the above criteria. In the case of ambiguity, the full‐length manuscript was reviewed to determine eligibility. Two individuals also performed a methodological quality assessment based on standardized criteria that were adapted from those used in prior systematic reviews [14, 15]. Results were summarized based on the domain of psychological intervention. This study was pre‐registered on the Open Science Framework (OSF) repository prior to study eligibility screening (https://osf.io/64fxy).

### 2.2 Definition of Positive Psychology Interventions

The concept of positive psychology interventions is broad and not well‐defined. Consistent with prior psycho‐oncology reviews [12, 16], positive psychology interventions were defined as psychological interventions that primarily aimed to enhance positive psychological resources such as well‐being, meaning, hope, resilience, life satisfaction, positive emotions, and personal growth, rather than solely targeting symptom reduction. Within this framework, positive psychology interventions encompassed a range of volitional activities designed to cultivate adaptive psychological capacities, which included mindfulness‐based approaches. Given the early stage of research on positive psychology approaches in prostate cancer, cross‐sectional and qualitative studies were included that examined psychological processes and coping‐oriented strategies [17] or aligned with positive psychology constructs (e.g., hope, resilience, mindfulness), as these constructs are commonly targeted within intervention frameworks.

### 2.3 Outcome Measures

Outcome measures varied across studies, including validated measurements for psychological distress and quality of life and positive psychological constructs such as mindfulness, post‐traumatic growth, and well‐being. Commonly used tools included the Hospital Anxiety and Depression Scale (HADS), Functional Assessment of Cancer Therapy–Prostate (FACT‐P), Life Orientation Test‐Revised (LOT‐R), European Organization for Research and Treatment of Cancer Quality of Life Questionnaire (EORTC QLQ), Center for Epidemiologic Studies Depression Scale (CES‐D), Brief Symptom Inventory (BSI), Intolerance of Uncertainty (IUS), Five Facet Mindfulness Questionnaire (FFMQ), PROMIS Global Health‐10 (PGH‐10), World Health Organization Quality of Life Scale (WHOQOL‐BREF), Memorial Anxiety Scale for Prostate Cancer (MAX‐PC), Mindful Attention Awareness Scale (MAAS), Adult Hope Scale (AHS), and measures of Post Traumatic Growth Inventory (PTGI).

## 3 Results

**FIGURE 1:**
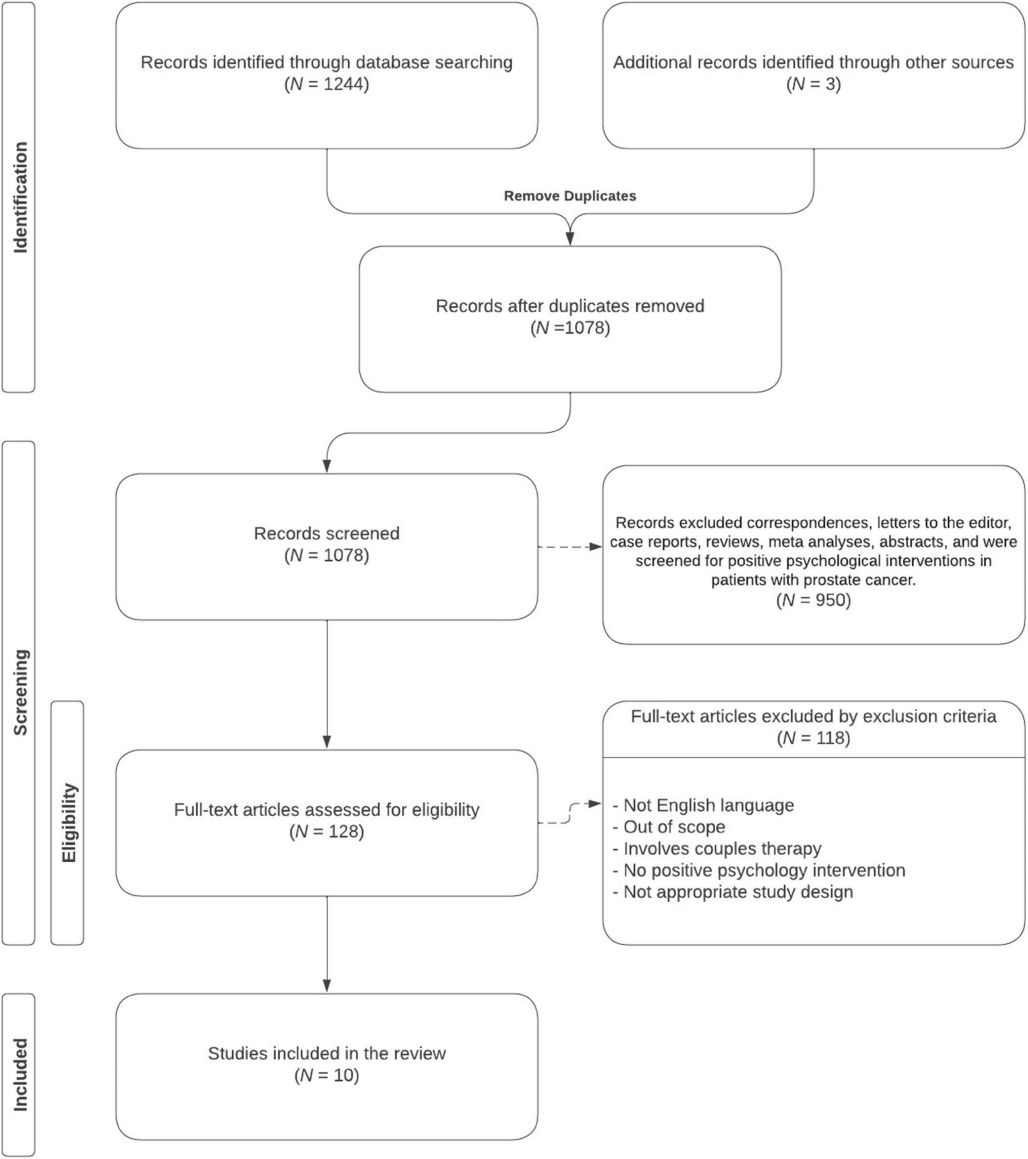
Flow diagram.

PubMed/Medline, Embase, and PsycInfo database searching resulted in 1078 bibliographic results. After removing duplicates and applying the filtering criteria, 10 studies met the inclusion requirements and were included in this review (Figure 1). Of the 10 included studies, 2 were randomized clinical trials [18, 19], 2 were pilot feasibility trials [20, 21], 3 were single‐arm interventional studies [22, 23, 24], and 3 were cross‐sectional studies [17, 25, 26]. Manuscripts focused on older adults, with reported mean ages ranging from 54.5 to 72.0. All manuscripts included patients with prostate cancer, but two included both breast and prostate cancers [22, 23]. On quality assessment, while most studies were prospective in nature and all studies clearly defined their population and outcome measures, few studies had a sample size of at least 100 or a follow‐up period of at least a year. The study characteristics are summarized in Table 1, and the quality assessment results are presented in Table 2.

**TABLE 1:** Summary of studies.

Study	Design	Inclusion/exclusion	Demographics	Objective	Intervention	Measure	Outcomes
Carlson et al. [23], Canada	Single Arm Cohort	Localized prostate or breast cancer. At least 3 months since surgery. Exclusion for (1) chemotherapy or radiation within 3 months, (2) mood, anxiety, or psychotic disorder, (3) autoimmune disorder, (4) prior participation in mindfulness‐based stress reduction (MBSR) program.	*N* = 59. Mean age 55, married: 71%. 49 women with breast cancer, 10 men with PCa.	Investigate the effect of a MBSR program on quality of life, stress, mood, lymphocyte counts, and cytokine production.	8‐week MBSR program (e.g., meditation, yoga, home practice). Periodic surveys, labs, and vitals.	Quality of life measures: EORTC, POMS, SOSI. Leukocyte subclasses (e.g., natural killer, helper T, cytotoxic T, B cells), and intracellular cytokines (e.g., TNF, INF‐gamma, IL‐4, IL‐10).	MBSR participation was associated with increased reported QOL and decreased symptoms of stress. There was no significant change in leukocyte subclasses, but there was an increase in IL‐4, and decrease in IFN‐gamma and IL‐10, which may be associated with a less depressive state.
Carlson et al. [22], Canada	Single Arm Cohort	Localized prostate or breast cancer. At least 3 months since surgery. Exclusion for (1) chemotherapy or radiation within 3 months, (2) mood, anxiety, or psychotic disorder, (3) autoimmune disorder, (4) prior participation in MBSR program.	*N* = 59. Mean age: 55, married: 42%. 49 women with breast cancer, 10 men with PCa.	Investigate the effect of a MBSR program on quality of life, stress, mood, endocrine, immune, and autonomic parameters.	8‐week MBSR program (e.g., meditation, yoga, home practice). Periodic surveys, labs, and vitals.	Quality of life measures: EORTC, POMS, SOSI. Measures for stress (cortisol), pro‐inflammatory cytokines (Th1), blood pressure.	MBSR participation was associated with increased reported QOL and decreased symptoms of stress. Cortisol, Th1 pro‐inflammatory cytokine, and blood pressure also decreased over time.
Ramachandra et al. [19], UK	RCT	Women with metastatic breast cancer and men with metastatic PCa with expected survival > 6 months. Exclusion for active psychiatric disorder or cognitive impairment.	*N* = 46. Mean age: 67; 22 women and 24 men recruited.	Develop brief, self‐administered psychologic interventions to improve well‐being among patients with cancer. Assessing for acceptability.	Well‐being interventions: (1) keeping a well‐being diary, (2) using a mindfulness audio CD, (3) planning a pleasurable activity.	Quality of life and stress scales: WHO Quality of Life Scale BREF, HADS, SOFAS, LOT‐R, TIPI. Qualitative interviews.	Brief well‐being interventions are acceptable and feasible for patients with cancer. Patients engaging in well‐being interventions had improvement in QOL scores on WHO QOL BREF.
Chambers et al. [21], Australia	Pilot, mixed method	Advanced PCa. Exclusion for cognitive impairment or psychiatric illness.	*N* = 19. Mean age: 67, married or in a relationship: 84%, average time since diagnosis 5.5 years.	Identify if a mindfulness‐based cognitive therapy group intervention would be acceptable and well‐received among men with advanced PCa.	Group mindfulness‐based cognitive therapy, 2 h weekly for 8 weeks. Implemented surveys and open‐ended interviews.	General psychological and cancer‐specific distress: HADS, IES‐R, MAX‐PC, EPIC, FACT‐P, FFMQ. Interviews at completion of program.	Intervention was acceptable and positively well‐received by participants. There were improvements in anxiety, avoidance, and mindfulness. Qualitative findings from interviews included positive group identification, acceptance of diversity, peer learning, and acceptance of disease progression.
Chambers et al. [26], Australia	Cross‐sectional	Metastatic or castration resistant PCa. Exclusion for history of head injury, dementia, current psychiatric illness, or concurrent cancer.	*N* = 190. Mean age: 71, married or partnered: 75%, average time since diagnosis: 6 years.	Examine extent that mindfulness skills influence distress and health‐related quality of life.	Survey about psychologic and cancer‐specific distress and quality of life.	General psychological and cancer‐specific distress: FACT‐P, FFMQ, BSI‐18, IES.	Non‐judgement of inner experience, non‐reactivity to inner experience, and acting with awareness were associated with higher health‐related QOL and lower psychologic distress.
Victorson et al. [20], USA	Pilot RCT	Men with localized PCa undergoing active surveillance.	*N* = 43. Mean age: 70, married: 83%.	Assess feasibility, acceptability, and preliminary efficiency of mindfulness intervention.	8‐week mindfulness intervention versus attention control arm (who received a mindfulness book).	Feasibility, acceptability measure: overall satisfaction with course, frequency of meditation. Preliminary efficacy based on PCa anxiety: MAX‐PC; personal stressors: PGTI, IUS; mindfulness: MAAS; mental health and physical health scores: PGH‐10.	Mindfulness training was generally feasible and acceptable. Over time, men in the mindfulness group had significant increases in posttraumatic growth compared to control arm.
Chambers et al. [18], Australia	RCT	Advanced PCa (metastatic and/or castration resistant). Exclusion for history of head trauma, dementia, psychiatric illness, or concurrent cancer.	*N* = 189. Mean age: 71, married: 76%, average time since diagnosis: 5.9 years.	Explore if mindfulness‐based cognitive therapy would reduce distress in men with advanced PCa.	8‐week group mindfulness‐based cognitive therapy (MBCT) versus usual care.	Psychologic distress: BSI‐18; cancer‐specific distress: IES, PSA anxiety subscale of MAX‐PC.	MBCT was not more effective than usual care in reducing distress in men with advanced PCa.
Zhao et al. [25], China	Cross‐sectional	Stage 1–2 PCa within 18 months of histologic diagnosis. Exclusion for pre‐existing psychiatric illness, intellectual impairments, or concurrent cancer.	*N* = 564. Mean age: 60, married or partnered: 88%. Stage I: 52.8%, Stage II: 47.2%.	Explore how perceived social support, hope, and resilience impact depression in men with PCa.	Questionnaires regarding depression, social support, hope, and resilience.	Questionaries about depression: CES‐D; perceived social support: MSPSS; hope: AHS; resilience: RS.	Perceived social support was negatively associated with depressive symptoms. Hope and resilience both significantly mediated this association.
Martin et al. [17], USA	Cross‐sectional	Self‐identified Black men with early to late‐stage PCa. Actively receiving cancer‐directed treatment or completed treatment. Exclusion for major psychopathology or cognitive impairment.	*N* = 95. Between age 60–69: 43%, married or living with partner: 64%.	Identify if African‐centered coping strategies and resilience are associated with lower psychological distress.	Questionnaires regarding distress and coping strategies.	Distress and depressive symptoms: HADS, MAX‐PC, CES‐D; coping strategies: ACSI.	African‐centered coping strategies were not associated with lower psychological distress, though greater resilience was associated with lower anxiety and depression.
Ilie et al. [24], Canada	Qualitative, Single Arm Cohort	Localized PCa, able to travel to Halifax, Nova Scotia on three separate occasions.	*N* = 30. Mean age: 69, in a relationship: 100%, greater than 2 years from diagnosis: 73%.	Determine if a patient empowerment program would promote mental and physical health for men with localized PCa.	28‐day PCa patient empowerment program (PC‐PEP), includes daily patient education, empowerment videos, prescribed physical activity.	Quantitative exit surveys (e.g., interest in program, likelihood to recommend PC‐PEP to others), and themes from semi‐structured focus group interviews.	Patients highly rated the program (9.6/10), especially the online, home‐based component. They expressed that the program helped reinforce confidence to adopt healthy lifestyle choices. Recommended early integration of these types of activities after diagnosis.

Abbreviations: ACSI, Africultural Coping Structure Inventory; AHS, Adult Hope Scale; BSI‐18, Brief Symptom Inventory; CES‐D, Center for Epidemiologic Studies Depression Scale; EORTC, European Organization for Research and Treatment of Cancer Quality of Life Questionnaire; EPIC, Expanded UCLA Prostate Cancer Index; FACT‐P, Functional Assessment of Cancer Therapy—prostate; FFMQ, Five Facet Mindfulness Questionnaire; HADS, Hospital Anxiety and Depression Scale; IES‐R, Impact of Event Scale‐Revised; IUS, intolerance of uncertainty; LOT‐R, life orientation test‐revised; MAAS, Mindful Attention Awareness Scale; MAX‐PC, Memorial Anxiety Scale for prostate cancer; MBSR, mindfulness‐based stress reduction; MSPSS, Multidimensional Scale of Perceived Social Support; PCa, prostate cancer; PGH‐10, PROMIS Global Health‐10; POMS, Profile of Mood States; PTGI, Post Traumatic Growth Inventory; QOL, quality of life; RCT, randomized controlled trial; RS, Resilience Scale; SOFAS, Social and Occupational Functioning Assessment Scale; SOSI, Symptoms of Stress Inventory; TIPI, Ten‐Item Personality Inventory.

**TABLE 2:** Quality assessment of studies.

	Carlson et al. [23]	Carlson et al. [22]	Ramachandra et al. [19]	Chambers et al. [21]	Chambers et al. [26]	Victorson et al. [20]	Chambers et al. [18]	Zhao et al. [25]	Martin et al. [17]	Ilie et al. [24]
Study population: describes patient population (e.g., demographics), inclusion and exclusion criteria	+	+	+	+	+	+	+	+	+	+
Study size: ≥ 100 participants	−	−	−	−	+	−	+	+	−	−
Study design: prospective study	+	+	+	+	−	+	+	−	−	+
Follow‐up period: follow‐up ≥ 12 months	−	+	−	−	−	+	−	−	−	−
Follow‐up completion: percentage of enrolled participants who did not complete survey, dropped out of study, or were lost to follow‐up ≤ 20%	−	−	−	−	+	−	−	+	+	+
Outcome measure: relevant outcome measure, description of how outcome measure was calculated	+	+	+	+	+	+	+	+	+	+
Analysis: Appropriate statistical analysis. Negative score if only correlation or qualitative findings reported	+	+	+	+	+	+	+	+	+	+
Quality score	4	5	4	4	5	5	5	5	4	5

### 3.1 Hope and Resilience Intervention

Two studies centered on hope and resilience in patients with prostate cancer [17, 25]. The results indicated that resilience and hope were significantly associated with lower levels of depression and anxiety [17, 25]. One study focused on African American patients [17], where resilience, but not coping strategies, was associated with both decreased anxiety and depression. The other study focused on Chinese‐speaking patients with early‐stage prostate cancers, and they found that support from hope and resilience levels were significantly associated with depressive symptoms based on the Center for Epidemiologic Studies Depression Scale [25].

### 3.2 Mindfulness‐Based Intervention

Mindfulness interventions were the most studied positive psychology interventions among patients with prostate cancer [18, 21, 22, 23, 26]. There are two types of mindfulness‐based interventions: mindfulness‐based stress reduction (MBSR) program and mindfulness‐based cognitive therapy (MBCT), an adapted version of the MBSR that explicitly focuses on turning negative thinking early on and preventing future depressive episodes.

#### 3.2.1 Mindfulness‐Based Stress Reduction Program

Among the mindfulness‐based intervention studies, two observational studies of the same population focused on MBSR efficacy; these studies included patients with both breast and prostate cancers, with only 10 patients who had prostate cancer. The results demonstrated that MBSR may significantly lower stress levels and improve quality of life in prostate cancer patients and also has a prolonged positive effect in lowering stress symptoms after 1 year of follow‐up [22, 23]. They also demonstrated a decrease in cancer‐related cytokine production.

#### 3.2.2 Mindfulness‐Based Cognitive Therapy

One observational study focused on patients with advanced prostate cancer (metastatic or castration‐resistant) and showed that three key mindfulness facets resulted in improved psychological distress and quality of life: awareness, non‐judgment, and non‐reactivity [26]. Another pilot trial on a similar population showed that administering MBCT group intervention resulted in a statistically significant drop in anxiety and avoidance as well as a non‐significant reduction in fear of cancer recurrence [21]. However, a full‐length trial by the same authors on 189 patients with advanced prostate cancer randomized to an 8‐week MBCT intervention compared to minimally enhanced care did not demonstrate that MBCT was effective in reducing distress in this population [18].

### 3.3 Meditation

Two studies mentioned meditation approaches, including recalling positive emotions and stress reduction techniques to improve the overall quality of life for patients with prostate cancer [20, 24]. One study describes a pilot trial where 24 men were randomized to a mindfulness meditation training program while 19 patients enrolled in an attention control arm. Both arms demonstrated significant increases in mindfulness, but the participants in the meditation arm also showed decreased anxiety and uncertainty intolerance, and increased posttraumatic growth and global mental health (PROMIS scale) [20]. The second study enrolled 30 patients with non‐metastatic prostate cancer to engage in a 28‐day empowerment program that involved meditation and stress reduction techniques. Participants expressed increased confidence to adopt healthy lifestyle choices and appreciation of the usefulness of the intervention; they also endorsed for its integration as a standard of care. However, findings were limited by the purely qualitative assessments, and possibly confounded by other interventions integrated with the program (physical activities, plant‐based diet, social connection, and support) [24].

### 3.4 Well‐Being Intervention

Well‐being interventions consisted of writing down positive thoughts, planning pleasurable activities, and other ways of provoking positivity [19, 24]. In a small clinical trial on patients with metastatic breast and prostate cancers, researchers found statistically significant improvements in quality of life (WHOQOL‐BREF) from a well‐being intervention focused on positive psychology techniques [19]. However, finding generalizability may be limited due to the small sample size and high attrition (25/46 completed 12 weeks and 10/46 completed 18 weeks). Well‐being methods were also included in the composite 28‐day intervention mentioned in the prior qualitative study above. Patients with prostate cancer gave positive feedback on well‐being interventions, such as recalling positive memories through a combination of meditation and breathing techniques [24].

## 4 Discussion

This comprehensive systematic review underscores that the primary positive psychology approaches advocated by Seligman, Rashid, Peterson, and Brown have seen limited exploration in men with prostate cancer. Among the varied list of described approaches, most studies in patients with prostate cancer fall into the categorizations of mindfulness‐based therapy, hope and resilience therapy, and well‐being therapy. These interventions were pursued because they also aimed to cultivate positive emotions, thoughts, behaviors, and cognitions, thus enhancing life satisfaction and sustainable happiness [10, 27]. Most studies showed a positive effect on improving outcomes, including psychological distress, depression, anxiety, quality of life, hope, happiness, positive engagement, positive emotion, and life satisfaction.

In this review, the mindfulness‐based responses appeared to have a more sustained happiness response compared to other approaches, which may be due to better adherence rates and superior improvements in quality of life. MBSR, developed by Kabat‐Zinn, was shown to reduce stress and anxiety in a variety of chronic conditions [28]. Through tools included in MBSR, such as meditation, patients could better handle negative emotions, reducing the inertia to start negativity [29]. Psychological stress plays a key role in activating the hypothalamic–pituitary–adrenal axis and sympathetic nervous system, leading to increased inflammation and immune dysregulation [30]. In the studies centered on MBSR on patients with prostate cancer, researchers found a drop in cancer‐related cytokine production after MBSR was administered [23]. These findings align with prior studies showing that mindfulness‐based interventions can influence immune‐related biomarkers and inflammatory signaling pathways such as through reductions in transcription factor NF‐kB, CRP levels, and pro‐inflammatory cytokines [31, 32]. Although the follow‐up study after 12 months was possibly confounded by cancer treatment, and the overall number of patients with prostate cancer was low [22], these preliminary biological findings provide a psychoneuroimmunological framework for the potential of mindfulness‐based positive psychology in this population.

Overall, mindfulness approaches have focused primarily on women and patients with breast cancers, where there is evidence for efficacy [12]. In this review, most studies were small but the one full‐sized randomized trial did not show a benefit to mindfulness on psychological or quality of life outcomes [18]. The potential factors for this are complex and unknown. Potentially, there are gender‐related distinctions and socially taught masculine preferences in help‐seeking, emotional expression, and overall engagement [33]. Additionally, as the study focused on older men with advanced cancers, perhaps there are generational barriers and less flexible cognitive awareness compared to younger patients or patients with early‐stage cancers.

The interaction of psychological responses and sex hormone biology may be especially pertinent to patients with prostate cancer, as common treatments such as androgen deprivation therapy (ADT) result in substantial decreases in testosterone levels. Testosterone suppression is known to affect psychological health including emotional regulation, changes in mood, and vulnerability to psychological distress [34]. Hormonal alterations have also been connected with systemic inflammation and depressive symptomatology, for example through increases in IL‐6 cytokine levels [35]. In this context, positive psychology interventions may help modulate psychological pathways affected by hormonal changes and help patients adapt to the psychological consequences of hormonal therapy.

Generally, most of these studies were limited by small sample size, qualitative findings, loss to follow‐up, and observational data. As reflected in the methodological quality assessment (Table 2), confidence in these findings is limited by heterogeneity in study design and overall rigor, which limits the ability to draw causal inferences. Additionally, not all patients benefited from the aforementioned interventions, indicating that positive psychology approaches may not be equally applicable to all patients with prostate cancer. Most studies also excluded patients with severe psychiatric disorders, who may potentially benefit most from these interventions. Moreover, the benefits of positive psychology outcomes may be difficult to quantify and historically have been observed in a more qualitative setting. As a result, these limitations mean that the current evidence should be interpreted as hypothesis‐generating rather than conclusive findings.

## 5 Conclusions

In conclusion, this systematic review demonstrates that positive psychology approaches may have benefit in patients with prostate cancer, but studies thus far have been small and mostly uncontrolled. Hormonal changes and inflammatory biomarkers may represent crucial pathways through which positive psychology interventions exert their effects on patients with prostate cancer. The positive aspects of psychological health may be particularly beneficial in this population where patients are particularly vulnerable to psychological distress. Further research should include larger prospective studies and clinical trials to determine whether these interventions have proven benefit in this understudied population.

## Author Contributions

Eleanor Xu wrote the main manuscript, reviewed the articles, and contributed to the study design. Quan H. Phung reviewed the articles and provided manuscript editing. Karie Runcie provided manuscript editing. Michael A. Liu supervised the project, contributed to the study design, reviewed the articles, and provided manuscript editing.

## Funding

The authors have nothing to report.

## Ethics Statement

The authors have nothing to report.

## Consent

The authors have nothing to report.

## Conflicts of Interest

Karie Runcie has consulting/advisory roles at Johnson & Johnson/Janssen. The consulting/advisory role has no impact on study design; data collection, analysis, interpretation; writing; and decision to submit. No other authors have potential conflicts of interest to disclose.

## Data Availability

Data sharing not applicable to this article as no datasets were generated or analysed during the current study.
